# Safety and immunogenicity of a first dose of SARS‐CoV‐2 mRNA vaccine in allogeneic hematopoietic stem‐cells recipients

**DOI:** 10.1002/jha2.242

**Published:** 2021-06-01

**Authors:** Patrice Chevallier, Marianne Coste‐Burel, Amandine Le Bourgeois, Pierre Peterlin, Alice Garnier, Marie C. Béné, Berthe‐Marie Imbert, Thomas Drumel, Steven Le Gouill, Philippe Moreau, Beatrice Mahe, Viviane Dubruille, Nicolas Blin, Anne Lok, Cyrille Touzeau, Thomas Gastinne, Maxime Jullien, Sophie Vanthygem, Thierry Guillaume

**Affiliations:** ^1^ Department of Hematology Nantes University Hospital Nantes France; ^2^ INSERM UMR1232 CRCINA IRS‐UN University of Nantes Nantes France; ^3^ Department of Virology Nantes University Hospital Nantes France; ^4^ Department of Hematology Biology Nantes University Hospital Nantes France

## Abstract

This was a monocentric prospective study testing the efficacy and safety of a first injection of BNT162b2 (Pfizer‐BioNTech) in 112 Allo‐HSCT patients. Antibody response to SARS‐CoV‐2 spike protein receptor‐binding domain was tested at the time of the second injection (Roche Elecsys). The study also included a non‐randomized control arm of 26 healthy controls. This study shows that a first dose of SARS‐CoV‐2 messenger RNA vaccine is safe and provides a 55% rate of seroconversion in allotransplanted patients compared to 100% for the controls (*p* < 0.001). Factors influencing the absence of response in patients were recent transplantation (<2 years), lymphopenia (<1 × 10^9^/L) and immunosuppressive treatment or chemotherapy at the time of vaccination.

## 1 INTRODUCTION

For more than one year, coronavirus disease 2019 (COVID‐19) has spread worldwide, responsible, as of spring 2021 for three million of deaths worldwide due to severe acute respiratory syndrome coronavirus‐2 (SARS‐CoV‐2) infection. Hope is finally emerging with the astonishingly fast development of various vaccine strategies. Among them, the efficacy and safety of SARS‐CoV‐2 messenger RNA vaccines has now been well documented in healthy populations [1]. Conversely, little is known of their administration in the context of immunocompromised patients mostly because they were obviously excluded from the initial trials testing this strategy.

Several studies have now reported that patients with hematologic malignancies who became infected by SARS‐CoV‐2 have poor outcomes, with a rate of mortality that may reach 33%–39% [2, 3, 4]. This is much higher than both in the general population with COVID‐19 and in patients with hematologic malignancies without COVID‐19. SARS‐CoV‐2‐related mortality in this group of patients is associated with higher age and the presence of comorbidities, but also with the type of hematological malignancy and of antineoplastic therapy. A recent study [5] has reported that 46% of patients with hematologic malignancy did not produce antibodies following mRNA vaccination and were therefore non‐responders. Patients with B‐cell chronic lymphocytic leukemia (CLL) were at a particularly high risk, as only 23% had detectable antibodies despite the fact that nearly 70% of these patients were not receiving any cancer therapy. Data are scarcer when considering allogeneic hematopoietic stem cell transplantation (Allo‐HSCT) recipients. If the death rate due to COVID‐19 infection can be as high as 25% [6], in this context, no data yet are available regarding the results of mRNA vaccination in such patients.

## 2 METHODS

This was a monocentric prospective study. The efficacy and safety of a first injection of BNT162b2 (Pfizer‐BioNTech) was evaluated in 112 Allo‐HSCT patients with no active graft‐versus‐host disease and more than 3 months after transplant, as recommended by French authorities. They had a median age of 57 years old (range: 20–75), and 45 were females. They were compared to 26 healthy controls (all belonging to the Hematology Department staff, median age: 52 years old [39–63], 22 females) included concomitantly. All participants received the vaccine between the January 20 and March 23, 2021 in the Hematology Department of Nantes University Hospital. None of them had a clinical history of COVID‐19. All participants provided informed consent, and the study was approved by the Nantes University Hospital review board.

Blood samples were collected before the first and second injections. Previous asymptomatic COVID‐19 infection was investigated in the first sample by testing for anti‐nucleocapside (N) antibodies (anti‐SARS‐CoV‐2 immunoassay, Roche Elecsys, Rotkreuz, Switzerland). Antibody response to SARS‐CoV‐2 spike protein receptor‐binding domain was tested at the time of the second injection (Roche Elecsys). As recommended by the manufacturer, titers ≥ 0.8 U/ml were considered positive. We did not use other assays for antibody response. Possible associations between clinical characteristics and antibody response were investigated using chi‐square and Wilcoxon tests with the R software via BiostaTGV. A *p* value < 0.05 was considered as statistically significant. Variable interactions were tested through multiple correspondence analyses (MCA) with XLSTAT (Paris, France).

Finally, all patients and controls were asked to complete a questionnaire to assess safety within the seven days following the first vaccination.

## 3 RESULTS

Patient characteristics are provided in Table 1. The median time from transplant to vaccination for the whole cohort was 22.1 months (range: 3–206). Previous asymptomatic infection was documented in four patients and one control through the presence of anti‐N antibodies. The median interval between the first vaccine and the serology assay was 21.5 days (range: 16–35) for patients and 23 days (range: 18–32) for controls. Sixty‐two patients (55.35%) were tested positive, while all controls (100%, *p* < 0.001) had a positive response. Among seropositive cases, median IgG titers were significantly higher in controls (35.1 U/ml, (2.2 → 250) than in patients (14.2 U/ml (0.9 → 250), *p* = 0.04). Considering the 10 patients < 6 months from the transplant, only one had a positive serology with a IgG titer of 3.1U/ml. When considering the 19 patients between 6 and 12 months from transplant, four had a positive serology (1.9; 2.9; 20.2 and > 250 U/ml).

**TABLE 1:** Patient characteristics

	All	Seropositive patients	
Characteristic	*N* = 112	*N* = 62 (55.35%)	*p* value
Median age: years (range)	57 (20–75)		NS
<40	18	12 (66.6%)	
40–60	46	25 (54.3%)	
≥60	48	25 (52%)	
Gender			NS
Male	67	34 (50.7%)	
Female	45	28 (62.2%)	
Median time from transplant to vaccination: days (range)	664 (91–6198)		<0.001
<12 months	29	5 (17.2%)	
12–24 months	35	19 (54.2%)	
>24 months	48	38 (79.1%)	
Underlying disease*			NS
Myeloid	75	40 (53.3%)	
Lymphoid	33	22 (66.6%)	
Donor type			NS
Geno‐identical	26	16 (61.5%)	
Matched unrelated	50	29 (58%)	
9/10 mis‐MUD	1	17 (48.5%)	
Haploidentical	35		
Conditioning			NS
Myeloablative	23	15 (65.2%)	
Reduced‐intensity	83	43 (51.8%)	
Sequential	6	4 (66.6%)	
Previous GVHD:			NS
Yes	57	29 (50.8%)	
No	55	33 (60%)	
Ongoing treatment**			0.001
No	81	53 (65.4%)	
Yes	31	9 (29%)	
Corticosteroids^$^	13		
Ruxolitinib	3		
Ciclosporin A	9		
Chemotherapy^$$^	6		
Lymphocyte count at time of vaccine: median (range) (x10^9^/L)	1.4 (0.15–9.9)		<0.001
<1 × 10^9^/L	34	8 (23.5%)	
≥1 × 10^9^/L	82	54 (65.8%)	

Abbreviations: GVHD, graft‐versus‐host disease; MUD, matched unrelated donor; NS, not significant.

* Acute myeloblastic leukemia *N* = 34; Myelodysplastic syndrome (MDS) *N* = 21; Myelofibrosis (MF) *N* = 8; MDS/MF N = 5; Chronic myelomonocytic leukemia *N* = 6; Blastic plasmacytoid dendritic cell neoplasm *N* = 1; Non‐Hodgkin lymphoma *N* = 17; Hodgkin lymphoma *N* = 4; Acute lymphoblastic leukemia *N* = 11; Multiple myeloma *N* = 1; Aplastic anemia *N* = 3; Porphyria *N* = 1/.

** immunosuppressive drugs or chemotherapy for relapse or relapse prevention.

^$^ Alone or in combination (ruxolitinib, Ciclosporin A, mycophenolate mofetyl).

^$$^ Three patients are receiving chemotherapy for lymphoma relapse (venetoclax +ibrutinib *n* = 1; methotrexate *n* = 1; tafasitamab+ revlimid *n* = 1) and three patients with AML 5′azacytidine in post‐transplant for relapse prevention.

**FIGURE 1:**
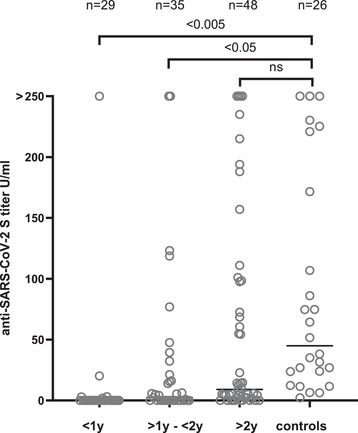
Anti‐SARS‐CoV‐2 S titers (with median value) after the first vaccine injection: comparison between patients for whom follow‐up after transplant was <1 year (y) versus between 1 and 2 years versus >2 years versus controls

Median IgG titers are given in Figure 1 according to the follow‐up of patients. The effect of immunosuppression is clearly visible, patients being at least at a 2‐year distance from Allo‐HSCT having similar rates of response as controls. Indeed, factors influencing the absence of response in patients were recent transplantation (<2 years), lymphopenia (< 1 × 10^9^/L), and immunosuppressive treatment or chemotherapy at the time of vaccination. This was confirmed by MCA that completely segregated seronegativity with lymphopenia, immunosuppressive treatment, and delay from Allo‐HSCT lower than 2 years and conversely seropositivity with normal lymphocyte counts, no treatment and Allo‐HSCT more than at least 2 years ago (data not shown).

This first vaccine injection appeared to be very safe both in patients and controls as only grade 1 or 2 adverse events were observed. Also, none of the participants required medical attention after the vaccination. Overall, reactions occurred in 47.8% of the patients and 66.6% of controls (*p* = NS). Only pain incidence was reported significantly more often in controls (62.5% vs. 20.2%, *p* = 0.0001), yet medication by paracetamol was not statistically different between both groups (Table 2).

**TABLE 2:** Vaccine‐related adverse effects within 7 days after the first dose in patients and controls

	Patients *n* = 94	Controls *n* = 24	*p* value
Any reaction^*^	45 (47.8%)	16 (66.6%)	NS
Injection‐site reactions			
Pain	19 (20.2%)	15 (62.5%)	0.001
Redness	5 (5.3%)	1 (4.1%)	NS
Swelling	6 (6.3%)	1 (4.1%)	NS
Systemic reactions			
Fever	1 (1%)	0	NS
Chills	7 (7.4%)	0	NS
Fatigue	19 (20.2%)	3 (12.5%)	NS
Myalgia	7 (7.4%)	1 (4.1%)	NS
Headache	12 (12.7%)	2 (8.3%)	NS
Nausea	2 (2.1%)	0	NS
**Medication (paracetamol)**	18 (19.1%)	8 (33.3%)	NS
**Medical attention required**	0	0	

Finally, none of the participants developed COVID‐19 infection between the first and the second vaccines.

## 4 DISCUSSION

This study shows that 55% of Allo‐HSCT recipients in this cohort developed anti‐SARS‐CoV‐2 S protein antibodies after a first injection of BNT162b2 vaccine. Conversely, all controls developed antibodies as expected [1]. This response after Allo‐HSCT appears to be higher than that reported in solid organ transplantation recipients (17%) [7], including renal transplantation (10.8%) [8], or patients with CLL [9]. The good response of Allo‐HSCT patients observed here also compares favorably with data obtained during the last H1N1 pandemic where several studies were performed in this population, showing a response rate comprised between 48% and 76% depending on the number of doses and whether a non‐adjuvanted or adjuvanted vaccine was used [10].

Allo‐HSCT recipients are thus confirmed to respond to vaccines yet at a lower extent than healthy individuals [11]. In fact, the response depends on a series of factors, especially the fact of being under treatment with chemotherapy/immunosuppressive drugs as well as the severity of immunosuppression at the time of vaccination. Moreover, as reported here and for organ transplants [7, 8], the timing after Allo‐HSCT is important for vaccine efficacy, patients vaccinated at distance from transplantation generally having better responses. Here, after 2 years, seropositivity was not statistically different between allografted patients and controls, suggesting that these patients can achieve a very good protection after vaccination.

Interestingly, data regarding the safety of the vaccine was also collected in our cohort. This first injection was safe as only grade 1 or 2 adverse events were reported. As for healthy individuals, frequently reported reactions included injection site pain, fatigue, and headache [12].

Data are still missing regarding the results of antibody response and safety after the second dose. These next results will be of crucial importance to decipher whether a third dose, especially within the 2 years following transplant, would be necessary to avoid complications and deaths due to COVID‐19 in such Allo‐HSCT recipients. It must, however, be noted that despite a suboptimal serological response, vaccination may provide clinical effectiveness through T‐cell responses. This has been explored in kidney transplant recipients, showing again poorer protection after 2 SARS‐CoV‐2 messenger RNA vaccination in this specific population [13].

Finally, other types of currently available vaccines or second‐generation vaccines should be explored in the future as well as the duration of protection in order to recommend or not annual vaccination for these patients.

## CONFLICT OF INTEREST

The authors declare no conflict of interest.

## AUTHOR CONTRIBUTIONS

Patrice Chevallier and Thierry Guillaume designed, performed, coordinated the research, analyzed, performed statistical analyses, interpreted the data, provided the figure, and wrote the manuscript. Marianne Coste‐Burel performed serology tests, generated the virologic data and commented on the manuscript. Marie‐C Bene performed statistical analyses and commented on the manuscript. Amandine Le Bourgeois, Pierre Peterlin, Berthe‐Marie Imbert, Thomas Drumel, Steven Le Gouill, Philippe Moreau, Beatrice Mahe, Viviane Dubruille, Nicolas Blin, Anne Lok, Cyrille Touzeau, Thomas Gastinne, Maxime Jullien and Sophie Vanthygem, provided data and commented on the manuscript.

## ETHICAL STANDARDS STATEMENT

All procedures followed were in accordance with the ethical standards of the responsible committee on human experimentation (institutional and national) and with the Helsinki Declaration of 1975, as revised in 2008.

## STATEMENT OF INFORMED CONSENT

Informed consent was obtained from all patients for being included in the study.

## ADDITIONAL CONTRIBUTIONS

We acknowledge the following individuals for their assistance with the study, none of whom were compensated for his or her contributions:

The Hematology Department nurses Patricia Lespart, Ghislaine Francois, and Katia Godart for administrating vaccines and their help in collecting samples and questionnaires, and the paramedical staff who participated in the study as controls.
